# Supplementary material for: Assessment of Genotoxicity in Human Cells Exposed to Modulated Electromagnetic Fields of Wireless Communication Devices
Source: Genes (Basel). 2020 Mar 25;11(4):347. doi: 10.3390/genes11040347 (PMC7230863; doi:10.3390/genes11040347)
Supplement: Supplementary file 1 [file genes-11-00347-s001.zip › genes-745982-R1_Supplementary Materials S1.pdf]

Supplementary Materials

Assessment of genotoxicity in human cells exposed to modulated electromagnetic fields of wireless communication devices

David Schuermann[1,*,#], Christina Ziemann[2,*,#], Zeinab Barekati[1], Myles Capstick[3], Antje Oertel[2], Frauke Focke[1], Manuel Murbach[3], Niels Kuster[3,4,ǂ], Clemens Dasenbrock[2,ǂ], and Primo Schär[1,ǂ]

[1] Department of Biomedicine, University of Basel, Mattenstrasse 28, CH-4058 Basel, Switzerland; david.schuermann@unibas.ch (D.S.), zbarekati@gmail.com (Z.B.), f.focke@dkfz.de (F.F.), primo.schaer@unibas.ch (P.S.)

[2] Fraunhofer Institute for Toxicology and Experimental Medicine ITEM, Nikolai-Fuchs-Strasse 1, D-30625 Hannover, Germany; christina.ziemann@item.fraunhofer.de (C.Z.), antje.oertel@item.fraunhofer.de (A.O.), clemens.dasenbrock@item.fraunhofer.de (C.D.)

[3] IT'IS Foundation, Zeughausstrasse 43, Zurich, Switzerland; capstick@itis.swiss (M.C.), manuel@murbach.eu (M.M.), kuster@itis.swiss (N.K.)

[4] Swiss Federal Institute of Technology (ETH), Zurich, Switzerland

[#] These co-first authors contributed equally to the work

ǂ These co-last authors contributed equally to the work

[*] To whom correspondence should be addressed: david.schuermann@unibas.ch (D.S.) or christina.ziemann@item.fraunhofer.de (C.Z.)

S1. Supplementary Material and Methods

S1.1 Cells and culturing procedures

All cell lines were grown and exposed under a controlled culture atmosphere of 37°C, 5% CO_2, and 95% humidity. Primary human MRC-5 lung fibroblast cells were obtained from ECACC at 25 population doublings. All exposure experiments were performed with cells at 37–43 population doublings. Cells were propagated and exposed in EMEM with α modification (Sigma-Aldrich, M8042) supplemented with 10% FCS (Sigma-Aldrich, F7524), 2 mM L-glutamine, MEM non-essential amino acids, and Penicillin-Streptomycin (60 U/mL-60 ng/mL). The immortalized human trophoblast cells, HTR-8/SVneo, generated by Graham [21] and provided by Dr Elena Fabbri [22], were propagated and exposed in RPMI-1640 medium (Invitrogen, #31870) supplemented with 10% FBS (Invitrogen, lot# 10270), 2 mM L-glutamine, and Penicillin-Streptomycin (60 U/mL-60 ng/mL). For exposure experiments, MRC-5 and HTR-8/SVneo cells were seeded in 35-mm Petri dishes (Nunc™, ThermoFisher Scientific) at 11–17 and 11–23 $\times 10^3$ cells/cm^2, respectively, 24 h prior to exposure start. Notably, standardized growth and propagation procedures, when possible even serum from the same production lot, were used to simplify inter-laboratory comparison, and to exclude any possible influence of the culturing method on the experimental outcome. Primary human fibroblast ES-1 (male, 6 years old) and HR-1d (male, 42 years old) cells were kindly provided by Dr Hugo W. Rüdiger (Vienna) and cultivated in DMEM supplemented with 10% FCS, 2 mM L-glutamine and Penicillin-Streptomycin (100 U/mL-100 ng/mL) (Sigma-Aldrich). Human osteosarcoma (U-2 OS) cells were cultivated in DMEM (Sigma-Aldrich) supplemented with 10% FCS (Sigma-Aldrich, F7524), 2 mM L-glutamine, and Penicillin-Streptomycin (60 U/mL-60 ng/mL). To obtain GFP-tagged XRCC1 cells, the coding sequence of XRCC1 (Genebank refseq NP_006288.2) was cloned in pEGFP-N1 (Invitrogen), and the plasmid was transfected into U-2 OS cells. Stable clones were selected and characterized for adequate expression levels of the full-length fusion protein.

S1.2 Exposure equipment

The radio frequency EMF exposure systems were built and provided by the Foundation for Research on Information Technologies in Society (IT'IS foundation), Zurich, Switzerland and are described in detail at https://itis.swiss/customized-research/emf-exposure-systems/in-vitro-sxc/. All wEMF exposure experiments were performed blinded for the experimenter; decoding and quality control was performed by the IT’IS foundation upon request after finalization of the data analysis.

S1.2.1 In-vitro exposure equipment for post exposure analysis

The sXc1950 exposure system (https://itis.swiss/customized-research/emf-exposure-systems/in-vitro-sxc/sxc1950/), equipped with an additional waveguide without antennas for control conditions, was used for bulk analysis of cultured cells. This setup is based on two R18 waveguides (wEMF exposed and sham-exposed) operating at 1950 MHz and positioned inside a commercial incubator to ensure constant environmental conditions. Six 35-mm Petri dishes per wave-guide placed in the H-field maxima were exposed simultaneously. The system provides an efficiency of > 50 W/kg per Watt input power, deviations from SAR uniformity of <30%, variability of <6%, and a temperature load of <0.03°C per W/kg average SAR. A computer-controlled signal and monitoring unit was developed to (1) generate complex modulated wEMF signals, (2) continuously monitor the field and environmental conditions (air temperature, fan cooling system), and (3) realize blinded exposure protocols.

S1.2.1 In-vitro exposure equipment for during exposure analysis

For live cell imaging under wEMF exposure, the sXcLive2450 system with an integrated microscope system was developed (https://itis.swiss/customized-research/emf-exposure-systems/in-vitro-sxc/sxclive2450/) for operation at 2450 MHz. State-of-the-art microscopic techniques, including confocal scanning and fluorescence microscopy, allow for live cell imaging during exposure. In contrast

to the classical approach of post analysis of cell responses, the instrument enables direct insight into the cell's response to EMF.

The system comprises a dual mode TE102 cylindrical cavity (Figure S1b) with quadrature feed and a computer controlled excitation system. Figure S1a shows the system block diagram; the computer is interfaced to the SMIQ03 signal generator (Rohde and Schwarz, Germany) via a GPIB connection and all other devices are connected via the universal serial bus (USB). These comprise a Measurement Computing USB-TEMP (Norton, MA) temperature-monitoring device, which can interface to thermistors and thermocouples and has 8 digital input – output lines (DIO), controlling the relays which set the exposure state. An M2i arbitrary waveform generator (AWG) (Spectrum Instrumentation GmbH, Germany) generates the I and Q waveforms to externally modulate the SMIQ03 for the WiFi and RFID signal generation. Finally a data acquisition (DAQ) system HS3 (TiePie, Netherlands) captures the detected waveforms and computes the rms exposure values.

As a proxy for the temperature of the cell medium, a temperature sensor is integrated into the cavity resonator of the live cell itself and two more in each of the RF power detectors, monitoring the forward and reflected power.

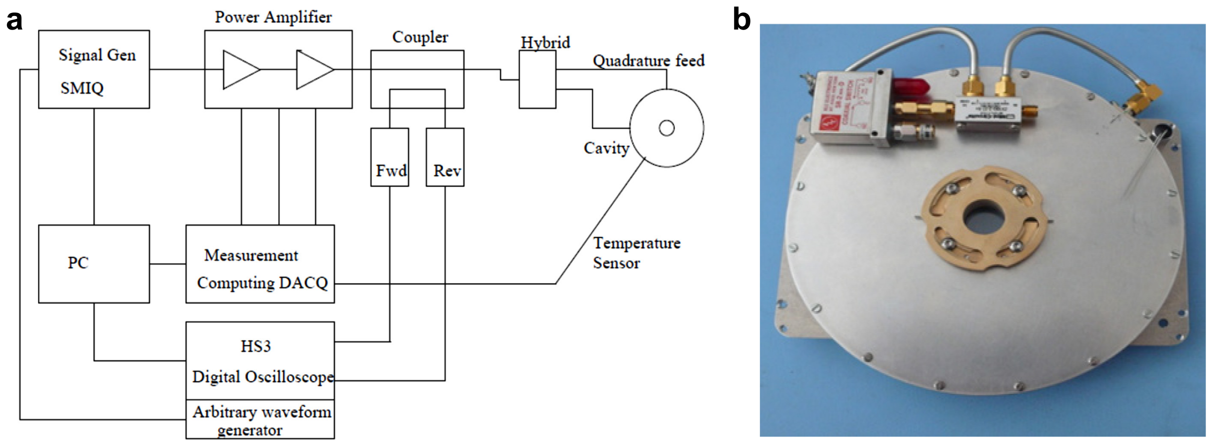

Figure S1. Live cell imaging system. (**a**) System block diagram and (**b**) dual mode cavity resonator of the sXcLive2450 system.

To achieve excellent SAR homogeneity and control, the system includes objective-lens-specific control settings that compensate for the close interaction of the exposure field and the lens. The 100× objective used the experiments herein has a working distance of only 0.3 mm from the cell monolayer or 0.1 mm from underside of the microscope 0.2 mm thick glass coverslip. For different positions of the x100 objective lens, we simulated SAR distributions (Figure S2).

The SAR can be characterized in terms of the average and standard deviation, plus any offset in average as the lens moves. If the range of lens movement is constrained to 6 mm diameter (this allows observation of a sufficiently large number of individual cells in the culture layer), the standard deviation of the SAR can be maintained at 0.41dB or 10% (Table S1). This is state of the art compared to the performance of reported *in vitro* exposure systems.

Experimental validation of the numerical results was performed using a DASY5NEO (SPEAG, Zurich, Switzerland) along with the smallest available SAR probe ET1D which is a 1 mm probe. Three planes were scanned in the media, 10×10 mm with 1 mm grid resolution, the extracted results for a vertical line through the center and at 3 mm offset are shown in Figure S3.

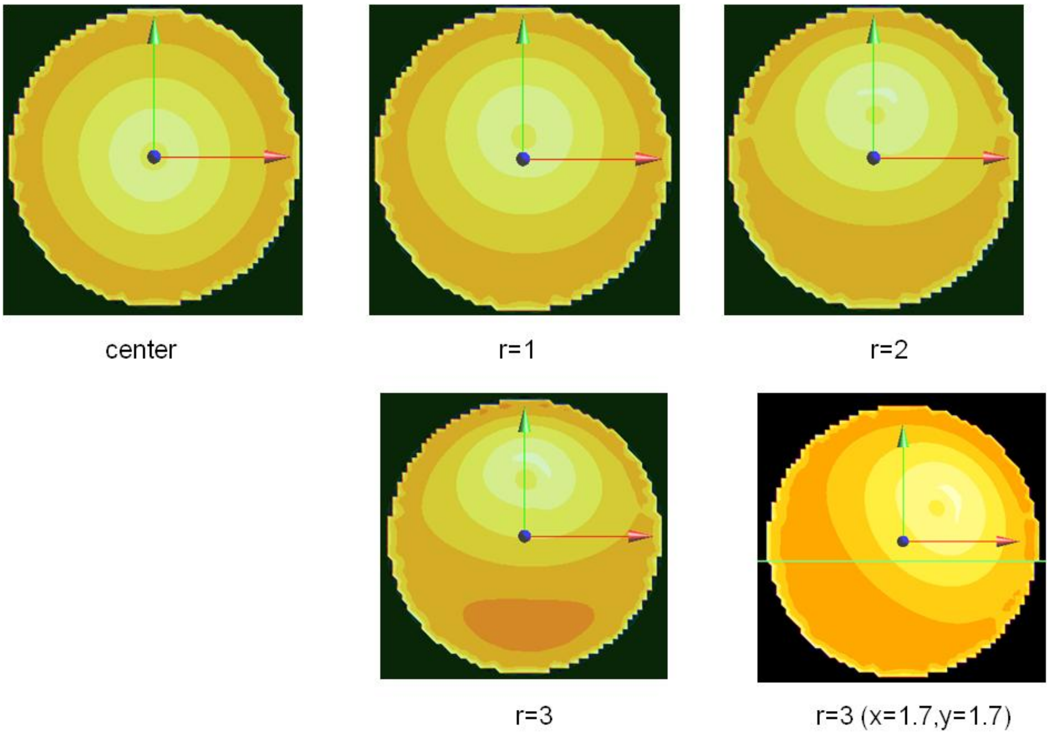

Figure S2. Simulated SAR distribution. SAR patterns in the cell monolayer for different lens positions of a 100× objective (1dB/contour)

Table S1. SAR variation and homogeneity. For a 100× objective lens, SAR average and variation was calculated as a function of observation point.

	**Radius over which SAR is characterized is 3 mm**		
lens position	average SAR (W/kg) per 1W input	standard deviation (dB)	difference to center (dB)
r=0	51.6	0.2	0
r=1	50.4	0.4	-0.1
r=2	47.0	0.6	-0.4
r=3	43.6	0.8	-0.7
r=3 (x=1.73, y=1.73)	45.2	0.6	-0.6
Overall	47.6	0.41	

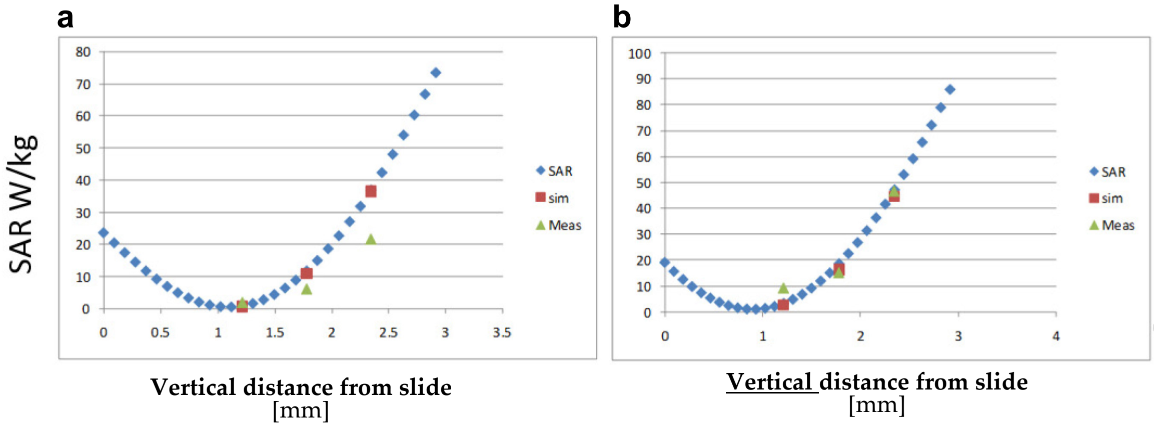

Figure S3. Experimental validation of numerical SAR. Measured and simulated SAR for 1 W input along a vertical line at (**a**) the center and (**b**) 3 mm offset.

S1.3 Signals and Modulation Characteristics

The field was applied either without modulation of the carrier wave signal or with modulation according to GSM, UMTS, WiFi, or RFID schemes, as described below.

S1.3.1 GSM

GSM signals were amplitude-modulated by rectangular pulses with a repetition frequency of 217 Hz and a duty cycle of 1:8, yielding frames of length 4.61 ms, each with a 576 μs burst. Since every 26th frame is idle, an 8 Hz modulation component was integrated into the signal. To save battery power during active periods when listening and not speaking into the phone, the number of active frames was reduced in discontinuous transmission mode (DTX). GSM talk modulation simulates a mobile-phone conversation by alternating between GSM mode and DTX mode.

S1.3.2. UMTS

Selection Rationale:

It was decided to maintain the UMTS signal as used with sXc1950 exposure setups (IT'IS Foundation Switzerland) that produced positive outcomes in previous studies [48]. The signal provides a realistic UMTS signal modulation (fast variation) and includes enhanced low-frequency components due to power control, frame, and channel definitions.

Signal Parameter:

The signal definition for the selected generic UMTS signal, displayed in Figure S4, consists of a WCDMA signal with amplitude modulation superimposed to create worst-case low-frequency power modulations that can be present in the UMTS communication system. A detailed description of the signal and rationale for selection of the low-frequency modulation is available in [27]. The measured frequency and time domain trace of the applied UMTS test signal are shown in Figure S5.

a

High frequency parameters	
Transmit frequency	1950 MHz
Signal bandwidth	5 MHz
WCDMA parameters	
Duplex mode	FDD
Chip rate	3.84 MHz
Number of data channels	6
Data channel bit rate	960 kbit/s
Data source	Pseudonoise, 2^{15} bit
Data channel spreading codes	$C_{4,0} - C_{4,3}$
Data channel weighting factor	1
Control channel structure	Pilot, TFCI, FBI, TPC bits
TFCI, FBI, TPC	all bits 1
Control channel spreading code	$C_{256,0}$
Control channel weighting factor	1
Scrambling code	$C_{long,64000}$
Pulse shaping filter	RRC, roll off 0.22
Low frequency parameters	
Signal periodicity	1 s
Inner loop power control	Step size 3 dB
Open loop power control	Step size 12 dB
Fading dynamic range	24 dB
Fading zigzag functions	every $1 - 1/2^n$ s ($n = 0, .., 6$)
Envelope ratio peak/average	27.7
Pulsed components	
Compressed mode sequence	3 slots idle, every fade
PRACH/PCPCH sequence	2 preambles, 2 frames data part

b

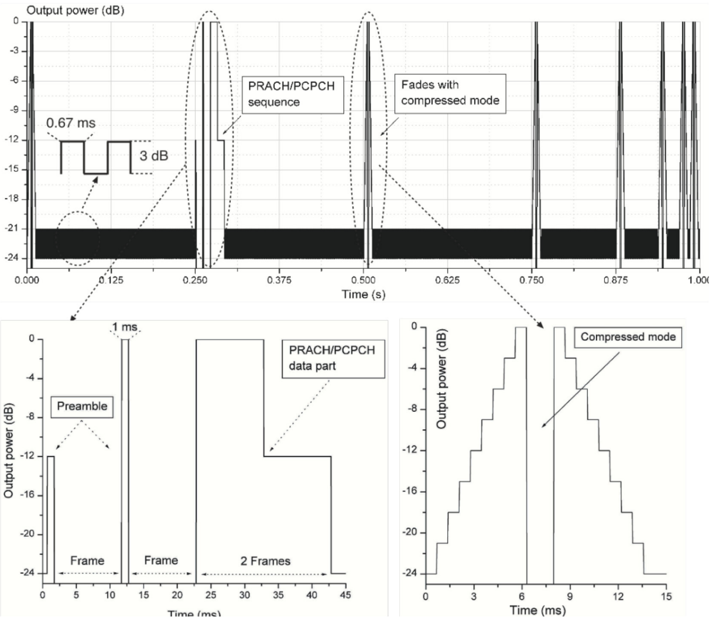

Figure S4. Generic UMTS signal. (**a**) UMTS signal definition. (**b**) Transmit power control envelope in the time domain. Adapted from [27].

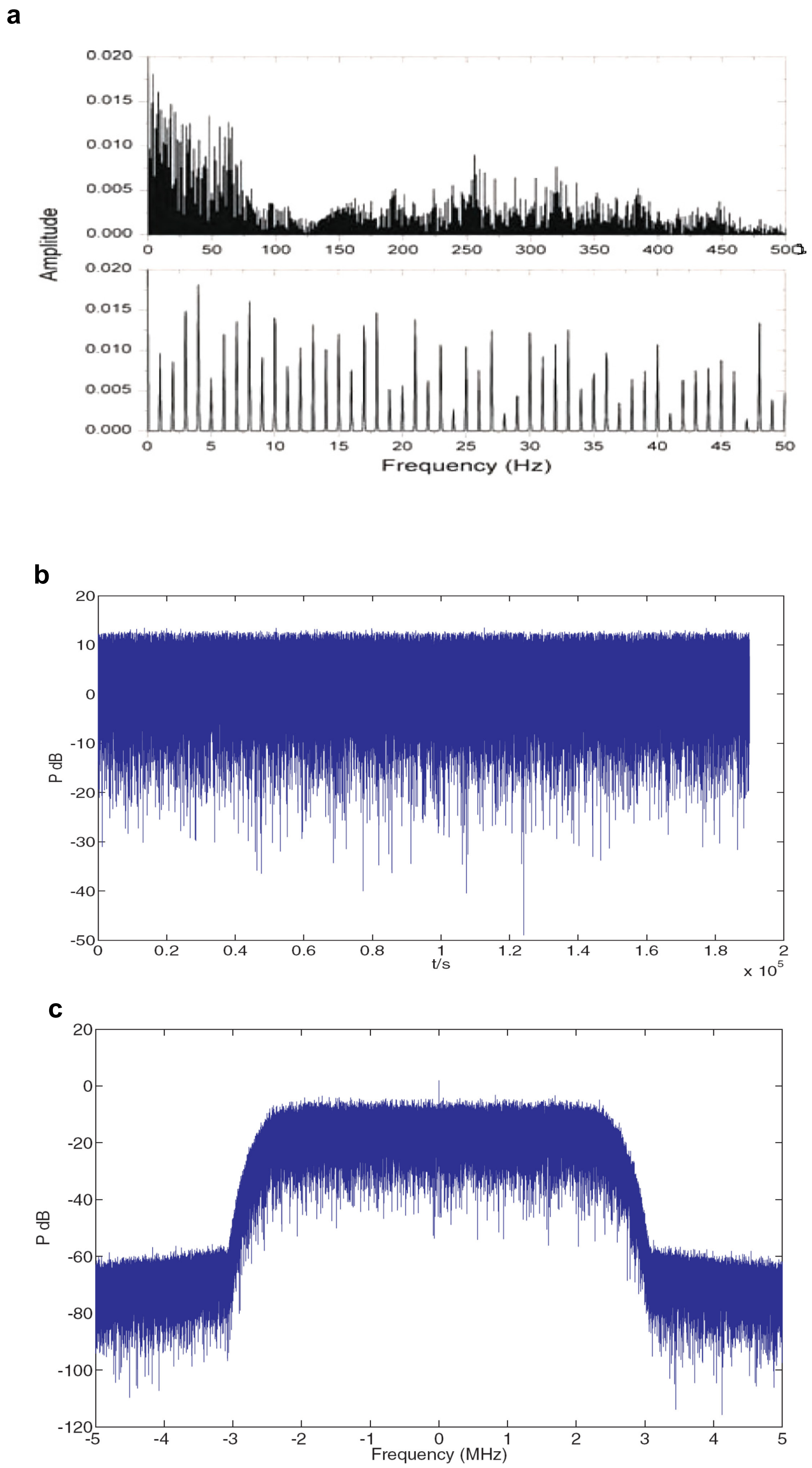

Figure S5. Characteristics of the UMTS test signal. (**a**) Frequency spectrum of the time-domain power envelope. (**b**) Time domain power envelope. (**c**) Frequency spectrum of the UMTS test signal. (Power control amplitude modulation deativated) [27].

S1.3.3 WIFI

Selection rationale:

A common feature of all managed WiFi (IEEE802.11x) networks is the transmission of a management beacon (Figure S6). The repetition rate of the beacon burst is often freely configurable, although typically a repetition rate of 100 ms is used. The different versions of the IEEE802.11x communication standard use a variety of different modulation techniques [26]. To reproduce the modulation features of the two most common modulation schemes – Differential BPSK (IEEE802.11b) and OFDM (IEEE802.11g/a) – it was decided to use data packets compliant with IEEE802.11g and including compatibility for IEEE802.11b. In this mode, the physical layer convergence protocol (PLCP) preamble and header are modulated according to the DBPSK scheme of IEEE802.11b, and the actual data in the physical layer service data unit (PSDU) is modulated according to the OFDM scheme of IEEE802.11g/a (Figure S7). As shown in Figure S8a, the BPSK preamble shows the 1 MHz and harmonic behavior of realistic IEEE 802.11b signals [28]. No such typical peaks are found in the demodulated spectrum of the OFDM54 PSDU (Figure S8b) or in other OFDM data rates. An example of the demodulated spectrum of an OFDM6 signal is shown in Figure S8c. The <-20 dBr bandwidths of both BPSK and OFDM signal types is <22 MHz, as shown in Figure S9.

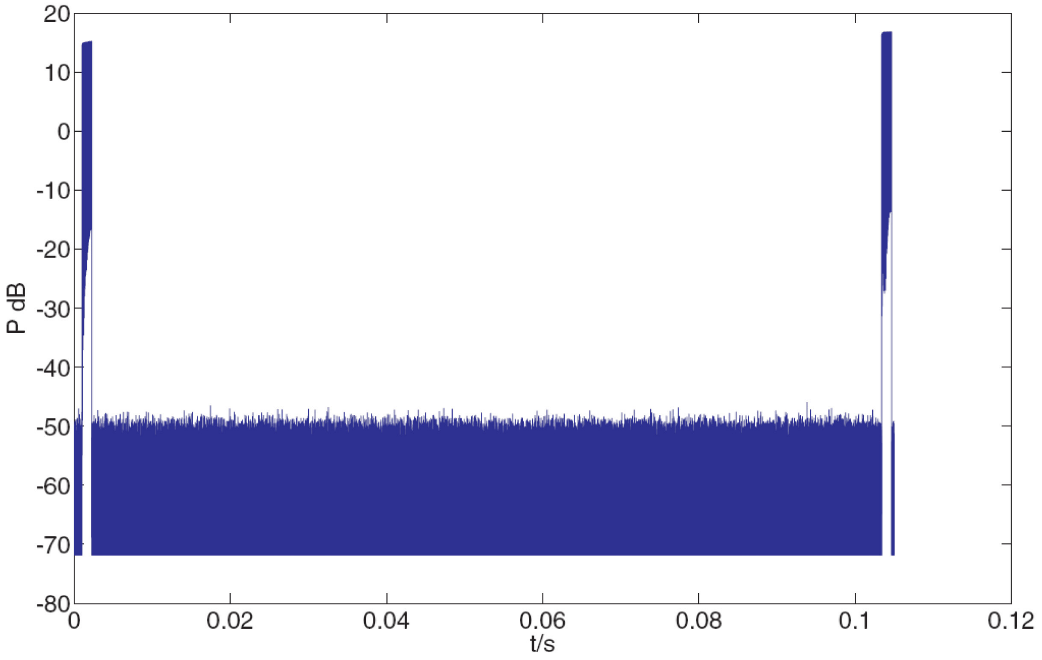

Figure S6. Time-domain signal of a WiFi base station transmitting an IEEE802.11b/g compatible beacon signal.

The probabilities of duty cycles and data packet lengths have been determined in field measurements [28]. The duty cycle T [%] is defined as the ratio of active duration t_{active} [s] to total duration t_{tot} [s] of the WLAN signal:

$$T = 100 \cdot \frac{t_{\text{active}}}{t_{\text{tot}}}$$

Figure 10a shows the cumulative distribution function (cdf) of the duty-cycle measured at the UGent – INTEC / IBBT offices (Ghent, Belgium). Measurements were executed at 33 different locations spread over the whole building. The median or 50th percentile (P50) is 2.5% and the 95th percentile is 2.7%. Figure 10b shows the cdf of the duty-cycle measured at 151 locations in Belgium, the Netherlands, and Stockholm, Sweden. The P50 is 1.4% and the 95th percentile is 11.1%. These duty-cycles might be worst-case estimates.

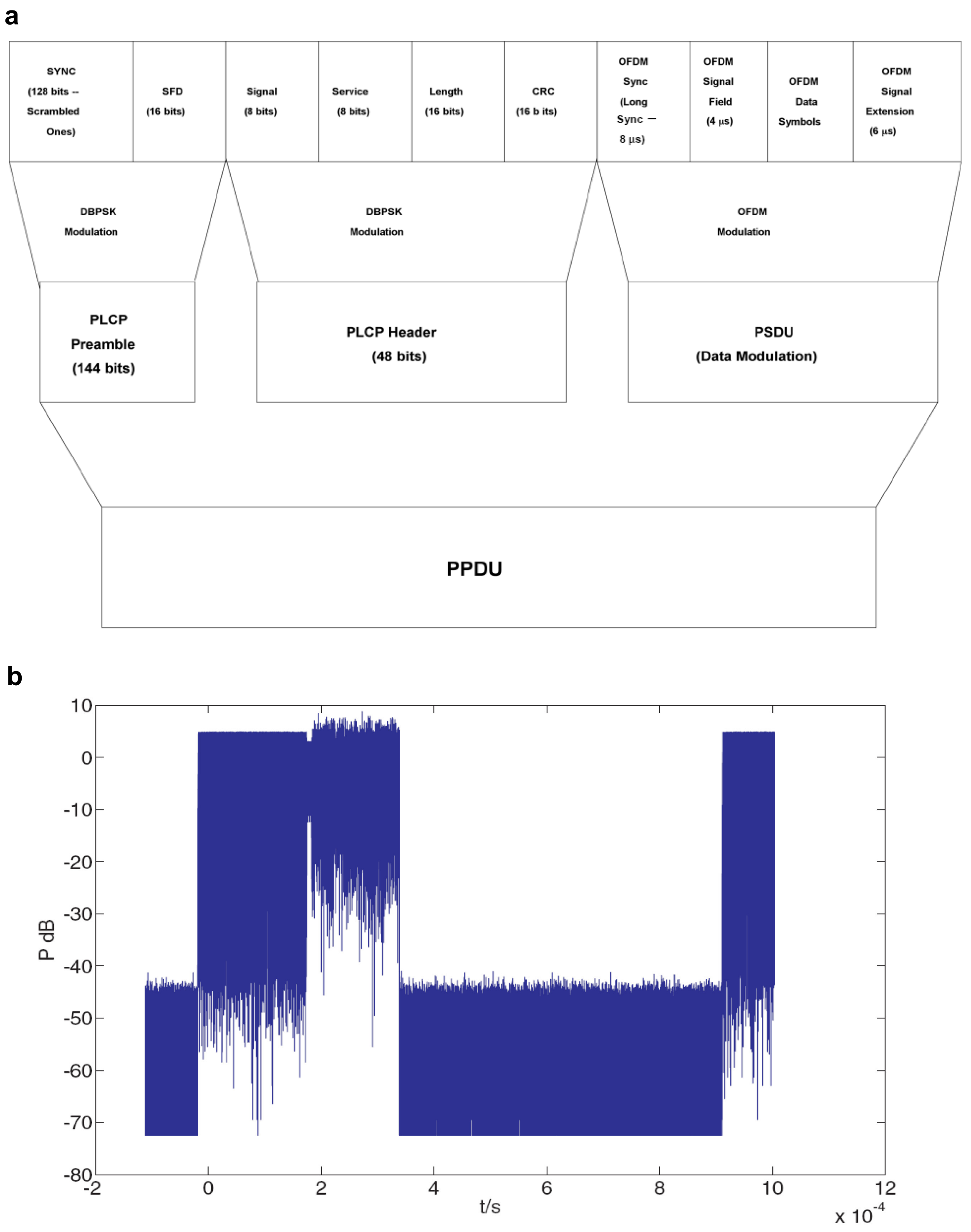

Figure S7. IEEE 802.11g physical protocol data unit format and signal. (**a**) Physical protocol data unit (PPDU) format with long preamble [29]. (b) IEEE802.11g signal with long BPSK preamble and 1000 byte PSDU data modulated according to the OFDM-54 scheme.

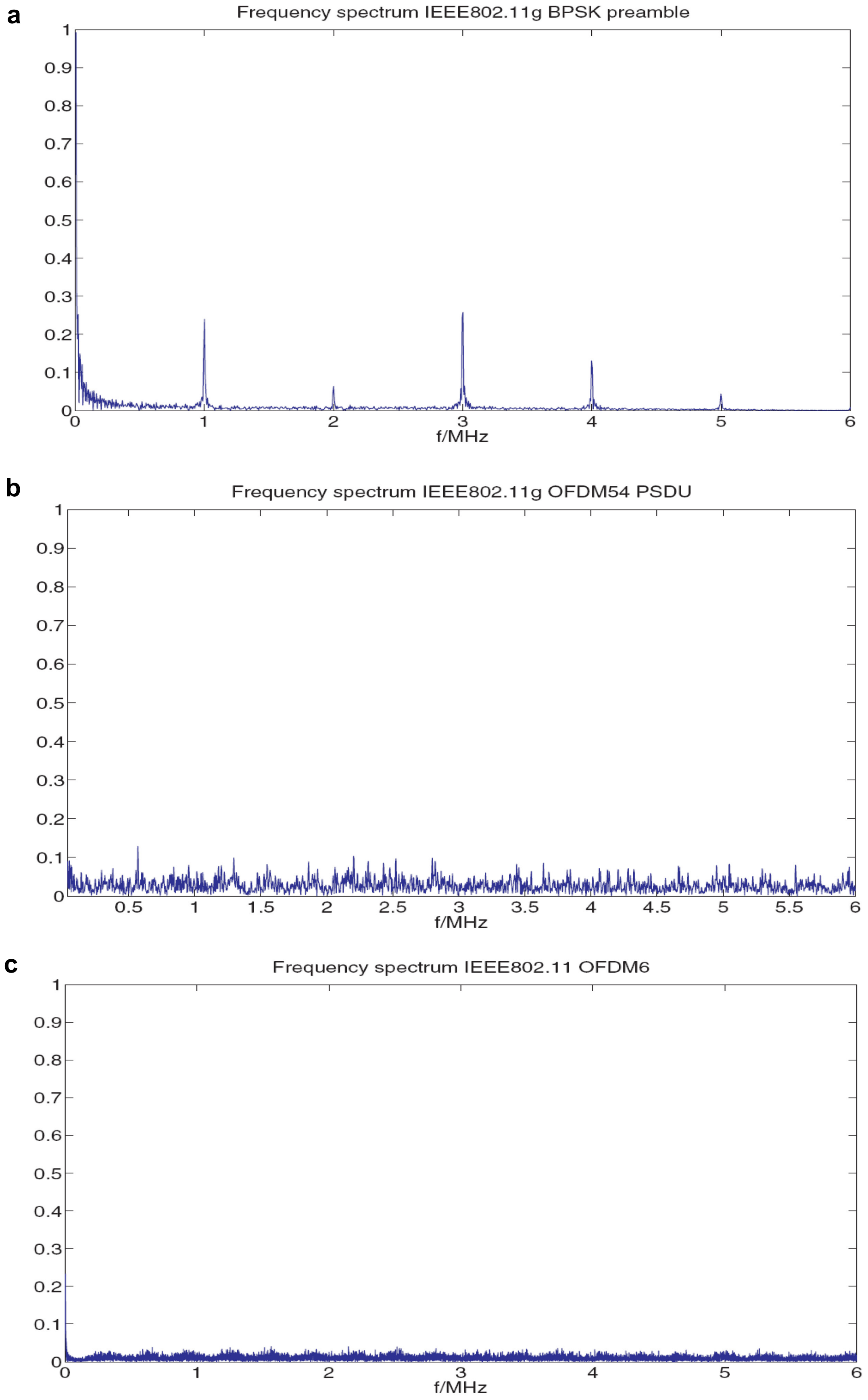

Figure S8: Frequency spectrum of the time-domain power variation: (**a**) of an IEEE802.11g BPSK preamble, (**b**) of an IEEE802.11g OFDM54 modulated PSDU, and (**c**) of an IEEE802.11g OFDM6 modulated PSDU.

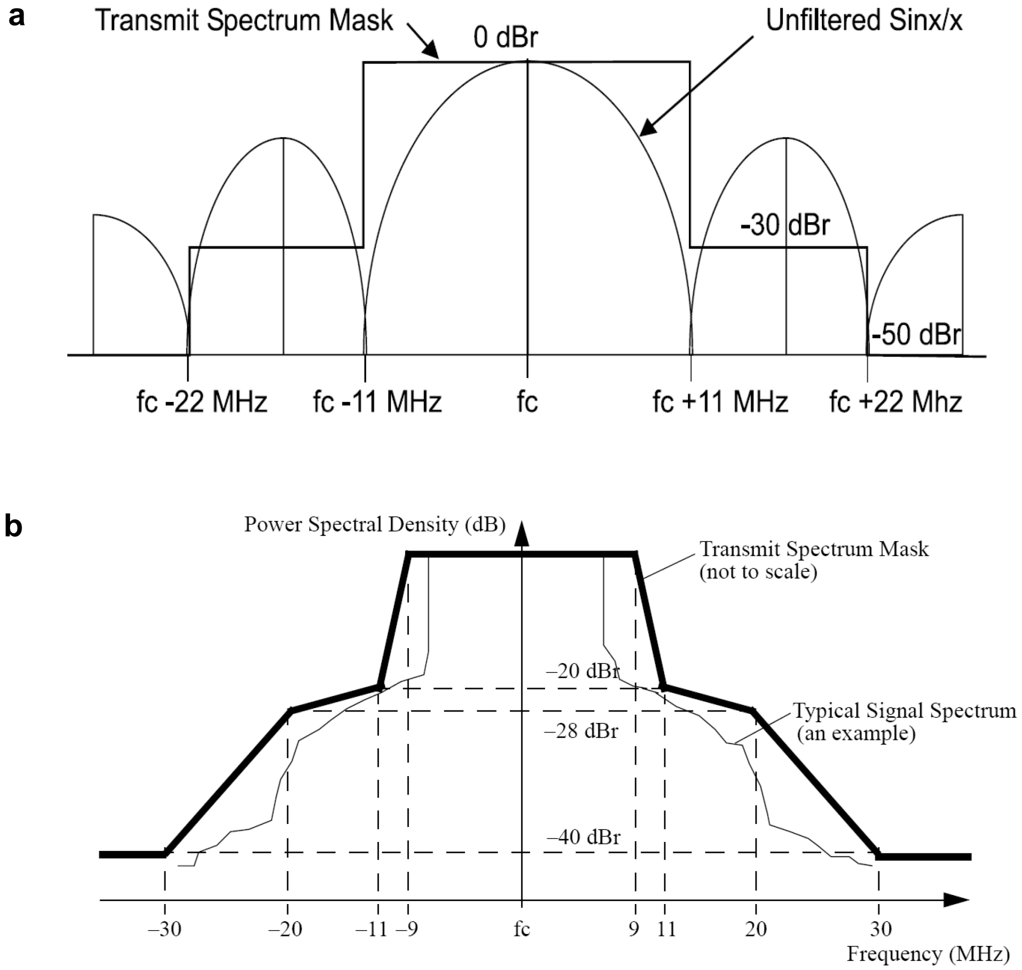

Figure S9. WiFi transmit spectral mask: (a) of IEEE 802.11b and (b) IEEE 802.11a

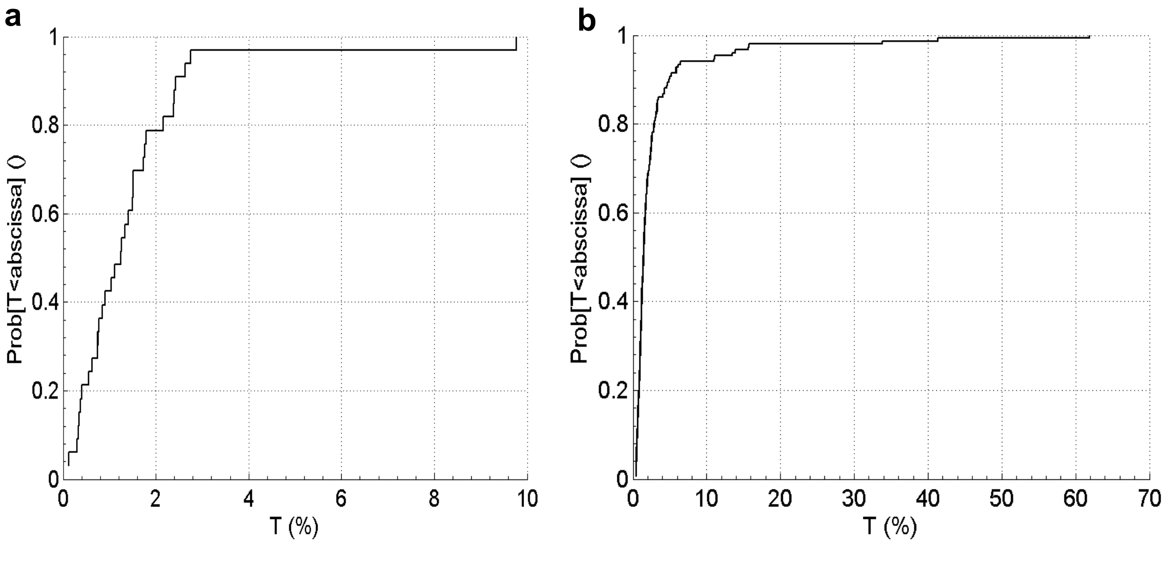

Figure S10. Cumulative distribution function of the duty-cycle T, measured (**a**) at the UGent-INTEC / IBBT office building or (**b**) at 151 locations in Belgium, the Netherlands, and Stockholm, Sweden.

Selected Signal Parameters

Based on the selection criteria previously presented, a signal consisting of repetitive 100 ms beacon transmissions is proposed. In addition to the beacon data, packets composed of a BPSK preamble and OFDM PSDU will be used to fill the 100 ms frame, to represent realistic duty cycles [28]. The signal characteristics for the 50th and 95th percentile duty cycles and the resulting requirements for the exposure systems are summarized in Table S2.

Table S2. WiFi signal characteristics and exposure system requirements.

**Signal characteristics:**			
Signal bandwidth		22	MHz
Beacon length		1.28	ms
Beacon frame rate		100	ms
Beacon duty cylce		1.3	%
Beacon burst PAR		3.0	dB
Data burst PAR (1000byte OFDM54 PSDU)		9.0	dB
Overall duty cycles (dc)	cumulative 50th percentile	1.4	%
	cumulative 95th percentile	11.1	%
**Exposure system requirements:**			
signal PAR	dc cumulative 50th percentile	27.5	dB
	dc cumulative 95th percentile	18.5	dB
signal dynamic range		>60	dB
signal bandwidth		>22	MHz
waveform sampling frequency		>44	MHz
waveform signal length		>100	ms

S1.3.4 RFID

Signal Parameters:

These parameters are based on the restrictions for RFID operation in Europe and represent a typical setup for passive UHF RFID, as this is considered a key technology for future pervasive computing [24,25]. Only the reader-to-tag communication is considered, as this has by far the strongest signal.

For testing, the effect of RFID signals on biological tissue the signal source with pulse interval encoding (PIE) (Figure S11) should be set up according to the parameters specified in Table S3.

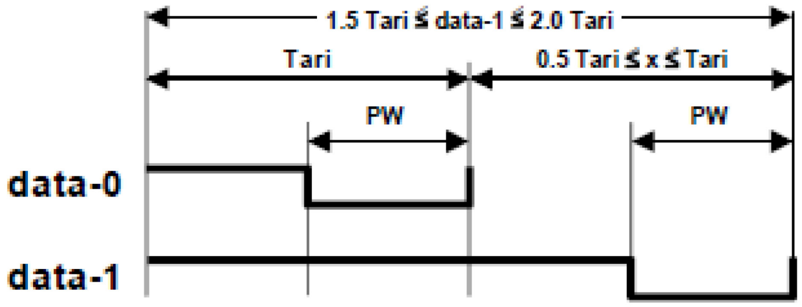

Figure S11. PIE symbols of RFID signal [25]

The sizes of the transmissions and their timing should also have characteristics similar to RFID, but the content can just be sequences of equiprobable data-0's and data-1's. The commands and timing for inventory operations are listed in Tables S4–S6 and Figures S12–S14.

Table S3. RFID signal characteristics.

Carrier Frequency:	866 MHz (865–868 MHz band)
Transmission Power:	33 dBm (EIRP)
Data Encoding:<br>Reference time: (Tari)<br>Time data-0:<br>Time data-1:<br>Pulse Width:	PIE (Figure S11)<br>8.5 µs<br>1*Tari<br>2*Tari<br>0.265*Tari [25] (p. 25)
Modulation:	DSB-ASK or SSB-ASK
Modulation Depth:	90% Nominal
Channel Bandwidth:	500 kHz
Channel Separation: (For dense-reader-mode)	*n* * 500 kHz, where *n*=1,2,...
Data Rate:	85 kbps [24] (p. 67)

Command Parameters:

These commands are based on the EPC Global Class 1 Generation 2 standard for communication with passive RFID tags. Only the commands for inventory tags have been taken into account, as this is the most common operation in an RFID network [25] (pp. 57–60).

Table S4. RFID command parameters

**Command:**	**Size:**	**Description:**
Preamble		Precedes a query command: (Figure S12)<br>12.5 µs + 1 Tari + 1 Rtcal + 1 TRcal<br>RTcal = time for a data-0 and a data-1=3*Tari<br>TRcal=1.1*RTcal<TRcal<3*RTcal
Frame Sync		Precedes all other commands than the Query:<br>12.5 µs + 1 Tari + 1 RTcal
Select	37 to 292 bits	
Query	22 bits	Command that initiates an inventory round, in order to identify the tags in the reader's proximity. In the command the reader specifies a frame size between 1 and 2^{15} slots. Each tag then chooses at random a slot to reply in. Their reply is a 16 bit random number.
Query Rep	4 bits	A command indicating the beginning of the next slot, and is used for traversing the frame
Query Adjust	9 bits	If the frame were traversed with tag collisions, the reader adjusts its frame size and asks the remaining tags to pick a new slot at random.
Ack	18 bits	When a tag reply, i.e. a 16 bit random number, is received, the reader acknowledges with a 2 bit command followed by the received 16 bit random number, in total 18 bits.
Nak	8 bits	Once acknowledged, a tag transmits the content of its memory protected with a CRC, if the CRC does not match at the reader it replies with a NAK

When simulating these commands it is reasonable to assume equiprobable data.

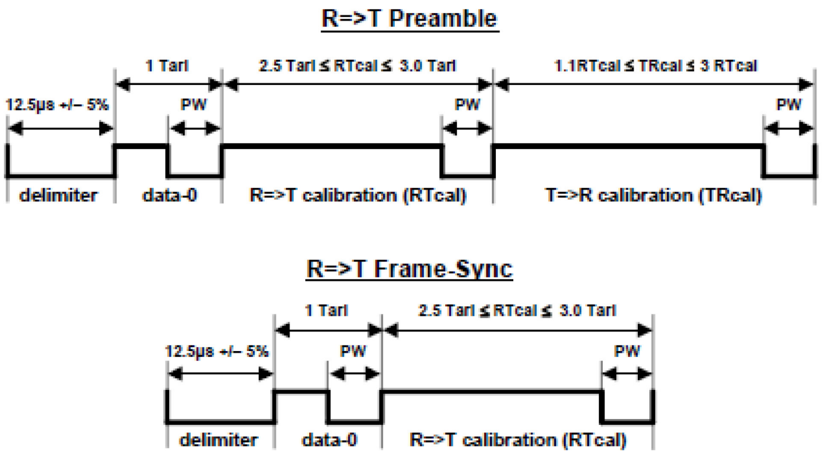

Figure S12. RFID R=>T preamble and frame-sync [25]

Timing Parameters:

The timing parameters when considering the commands used for inventorying are given in Table S5 [25] (p. 36):

Table S5. RFID response times

	Nominal Value:	Description:
T_1	max(RTcal,10*T_{pri})	Tag response time
T_2	Btw 3.0 and 20.0 times T_{pri}	Reader response time
T_3	$T_4 – T_1$	Time an interrogator must wait after T_1 before it issues another command
T_4	2.0*RTcal	Minimum time between reader commands

When considering the reader side of the communication link, only T_4 is of any interest.

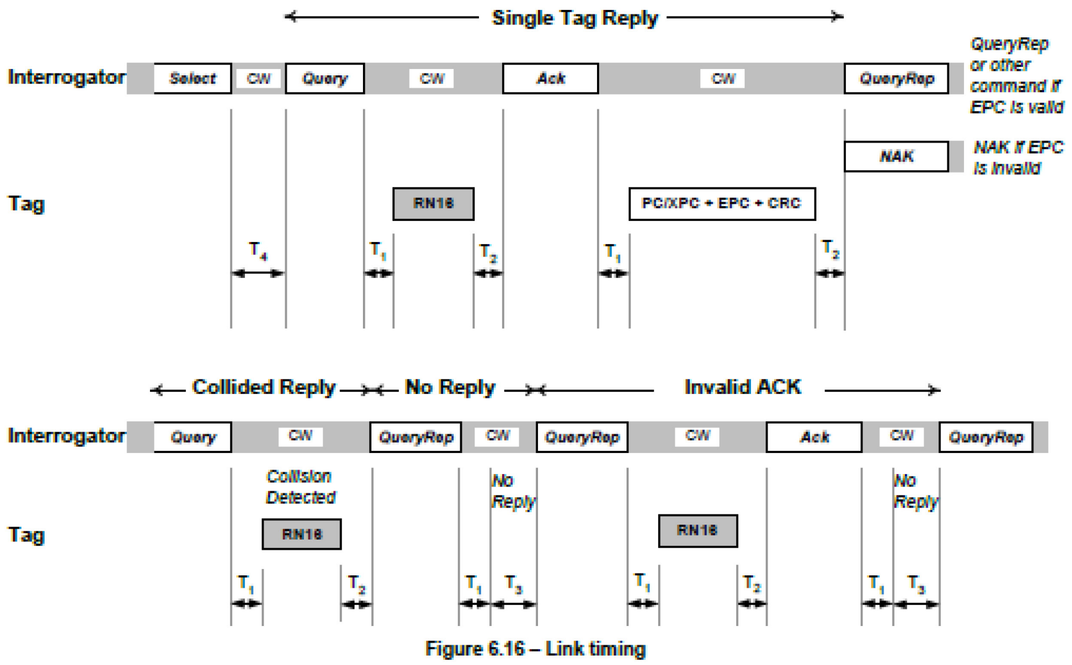

Figure S13. Link timing of RFID signal [25].

Power Template:

The interrogator power-up RF envelope should comply with the scheme in Figure S14 and the parameters listed in Table S6. Once the carrier level has risen above the 10% level, the power-up envelope should rise monotonically until at least the ripple limit M_l. Readers must not transmit any commands before the end of the settling time. Figure S14 shows the power template of the reader signals, and the parameters are explained in the following table [25] (p. 26):

Table S6. RF envelope parameters of RFID signal

	Nominal Value:	Description:
T_r	500 µs	Rise time
T_s	1500 µs	Settling time
M_s	1% of full scale	Signal level when off
M_l	5% of full scale	Undershoot
M_h	5% of full scale	Overshoot
T_f	500 µs	Fall time

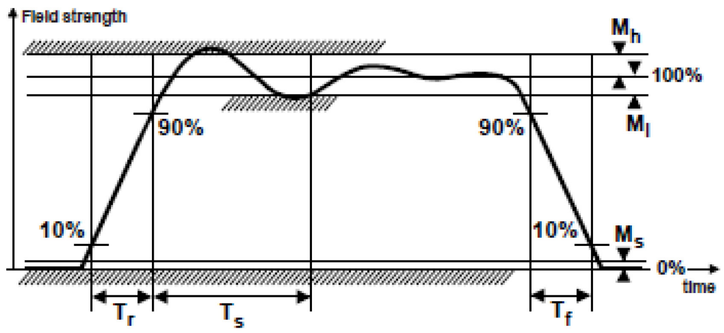

Figure S14. Interrogator power-up and power-down RF envelope [25]

Repetition Rate:

The standard does not specify any maximum repetition rate, but leaves it up to local regulations. Hence, the worst possible case, i.e., where the requirement is the minimum time interval between reader commands, should be considered. The default frame size of many commercial readers is set to 16 (2^4) slots, where the maximum frame size is 2^{15} slots. If a frame size of 16 is considered, an inventory round would give the following sequence of reader commands:

1. Select + the initial request
2. Follow with a sequence of 15 Query Rep's separated by the correct time intervals. Note that in these time intervals the reader is not quiet, but continuously transmits the carrier wave to keep tags powered up and enable them to reply (Figure S13). (To emulate that a tag has replied successfully and the reader replies with an Ack, slots could be chosen according to a binomial distribution, and after that particular Query Rep transmit an Ack.)
3. Wait T_4 seconds until a new round can be initiated with a new Select+Request

Therefore, such an inventory round was "recorded" and transmitted repeatedly to correspond to the scenario where a reader is continuously requesting tag replies, as would be the case in an actual RFID system. To avoid periodic transmission of the same command sequence, different frame sizes were used (only 2^i, where $i = 0.1, \ldots ,15$), and Ack commands were included randomly between the Query Rep commands.

S1.4. Alkaline Comet assays

Alkaline Comet assays were performed, according to Singh *et al.* [30], with some laboratory-specific modifications. As the main endpoint, "% DNA in tail", also called "Tail intensity", of at least 100 nuclei was determined per slide. As a direct measure of the amount of broken DNA, it can be standardized among various studies, laboratories and analyzing systems, and is linear over a wide range. So-called "hedgehogs" (overlapping nuclei/comets) were excluded. Depending on the Comet assay and scoring methods, the following variations in the protocol were applied:

S1.4.1 Laboratory 1

Induction of DNA strand breaks, alkali labile sites, and oxidative DNA base modification 8-hydroxydeoxyguanosine (8-OH-dG) was analyzed by the alkaline Comet assay, according to Singh *et al.* [30], or the human 8-oxoguanine DNA N glycosylase 1 (hOGG1)-modified version described by Smith *et al.* [33]. MRC-5 and HTR-8/SVneo cells were assessed after 1, 4 and 24 h of wEMF/sham exposure or upon incubation with the positive controls ethyl methanesulfonate (EMS; 0.25–0.75 µL/mL) or potassium bromate ($KBrO_3$; 0.5–1 mM). After wEMF exposure, cells were detached by trypsinization, counted by the CASY 1 cell counter (Model TT, Roche Innovatis AG). All steps following cell detachment were done under red light in order to avoid unspecific DNA damage due to UV-irradiation. Per treatment condition, two aliquots of 3×10^5 cells were transferred in 1.5 mL reaction tube and pelleted at 900 rpm for 5 min (Heraeus Biofuge 15, Thermo Scientific). Cells were then re-suspended in 80 µL of 0.75% (w/v) pre-conditioned low-melting-point agarose (LMA, Sigma-Aldrich) and applied to slides with roughened surface (Menzel-Gläser), which had been pre-coated with 0.5% (w/v) of normal-melting-point agarose (NMA, Sigma-Aldrich). The gels were overlaid with a cover slip, allowed to set at 4°C, before adding an additional layer of 100 µL 0.75% LMA, which were again covered with cover slips and transferred to 4°C. After removing the cover slips, the slides were incubated in lysis solution (2.5 M NaCl, 100 mM Na_2EDTA, 10 mM Tris-HCl, 8 g/L NaOH, 1% Triton-X100, 10% DMSO) overnight at 4°C. To remove the lysis solution, the slides were immersed three times for 5 min at room temperature, either in electrophoresis buffer (300 mM NaOH, 1 mM Na_2EDTA, pH >13) or enzyme buffer (40 mM HEPES pH 8.0, 100 mM KCl, 0.5 mM Na_2EDTA, 0.2 mg/mL bovine serum albumin) for alkaline or hOGG1-modified Comet assays; respectively. In the case of the hOGG1-modified Comet assays, one of the two slides per treatment condition was incubated with 0.16 U of human hOGG1 (New England Biolabs) in 100 µL of enzyme buffer at 37°C in a humidified atmosphere for 10–12 min. The second slide was treated with enzyme buffer only. Comparing hOGG1- with enzyme buffer-treated slides, increase in "% DNA in tail" is indicative for the occurrence of the oxidative base lesion 8-OH-dG. Placed in quadriPERM dishes (Greiner Bio-One), incubation was performed under a coverslip. For both the alkaline and the hOGG1-modified comet assay, slides were subsequently placed in a pre-cooled electrophoresis tank and covered with pre-cooled electrophoresis buffer. DNA was allowed to unwind for 20 min before electrophoresis at 24 V/300 mA for 20 min. Finally, slides were removed, neutralized by three changes of neutralizing buffer (0.4 M Tris-HCl pH 7.4) and stained with 80 µL of an ethidium bromide solution (20 µg/mL; Merck-Millipore) per slide. Finally, DNA damage was analyzed on an Axioskop fluorescence microscope (Carl Zeiss) by the Comet Assay III software (Perceptive Instruments). For each wEMF signal and treatment condition, at least three independent experiments were performed, determining the "% DNA in tail" of at least 100 nuclei per slide. The arithmetic mean of the single cell data was calculated per slide. Means of at least three independent exposure experiments were subjected to statistical analysis with the SigmaPlot software (Systat Software GmbH) applying Student's *t*-test for unpaired or paired values, as indicated. Difference were considered statistically significant at $p \leq 0.05$.

S1.4.2 Laboratory 2

Automated scanning and scoring – Metafer CometScan. Menzel SuperFrost Plus microscope slides were prepared by washing with EtOH, coating with 1% agarose/ddH_2O (BioRad), and air-drying. Immediately after the exposure cycles were finished, cells were harvested by trypsinization, washed

once with PBS, and resuspended in cold PBS at a density of 3 ×10^6 cells/mL. Then, the cell suspension was carefully mixed with 9 volumes of freshly prepared 0.75% low-melting-point agarose (SeaPlaque GTG, Lonza) in PBS and embedded on a slide pre-coated with a layer of the same agarose. Finally, another layer of agarose was added; each layer was allowed to solidify protected from light at 4°C for 20 min. Slides were then sequentially submerged in ice-cold Comet lysis buffer (Trevigen) for 45 min and 950 mL freshly-prepared electrophoresis buffer (1 mM Na_2EDTA, 200 mM NaOH, pH > 13) for 30 min. After DNA unwinding in the Trevigen Comet electrophoresis apparatus, electrophoresis was performed at 21 V for 25 min. Finally, slides were fixed by incubating in 250 mM Tris-HCl pH 7.6/ 90% MeOH for twice 10 min, washing with 50 mM Tris-HCl pH 7.6/ 80% EtOH and with 80% EtOH for 5 min, and air-drying. For the analysis of the Comet assays, slides were rehydrated with 10 mM Tris-HCl pH 8 for 2 × 15 min, and then mounted with the DNA staining solution; 10 mM Tris-HCl pH 8, 20% VectaShield (Vectorlabs)/ 5% DMSO/ 1x SYBRGold (Invitrogen). After incubating the slides for at least 2 h, automated scanning and analysis of the Comet assays with the Metafer CometScan (MetaSystem) system was performed. Post-analytic manual quality control was routinely performed to remove duplicates and wrongly recognized Comets. For each experimental condition, about 500 nuclei each from two technical replica gels were analyzed and combined for statistical analysis of the Comet parameter "% DNA in tail". Routinely, mean as well as median Comet parameters of at least three independent exposure experiments were analyzed for statistically sound differences with Student's *t*-tests (paired and unpaired as indicated) in MS Excel as well as with ANOVA and the indicated post-hoc pairwise comparison tests in GraphPad Prism 8.

Visual scoring – Directly after exposure, cells were harvested, counted, and resuspended in PBS at a density of 2.0 ×10^6 per mL. All steps after exposure were performed under dimmed light at 4°C. After mixing 1:9 volumes of the cell suspension with 0.5% low-melting-point agarose (Cambrex) pre-warmed to 37°C, 10^4 cells were applied to microscope slides pre-coated with 1.5% normal-melting-point agarose (BioRad). After solidifying the embedded cell layer for about 15 min under a cover slip, the cover slip was removed and another layer of low-melting-point agarose was applied. Slides were then immersed in freshly prepared ice-cold lysis buffer (2.5 M NaCl, 100 mM Na_2EDTA, 10 mM Tris-HCl pH 10, 1% Triton X-100, 10% DMSO) for 90 min, washed in ddH_2O, drained, and placed side-by-side in a gel electrophoresis tank. Slides were submerged with freshly made electrophoresis buffer (1 mM Na_2EDTA, 300 mM NaOH, pH >13) and incubated for 30 min to allow DNA unwinding prior to electrophoresis at 25 V/300 mA for 20 min. After electrophoresis, slides were washed with 0.4 M Tris-HCl pH 7.5 for 3 × 10 min, followed by fixation with EtOH absolute (2 × 5 min) and air-drying. Finally, nuclear DNA was stained with 20 µg/mL ethidium bromide, and analyzed with a fluorescent microscope at 400× magnification. Data collection and scoring was performed according to Anderson *et al.* [31] with minor modifications by Ivancsits *et al.* [32]. On two replicate slides for each individual exposure condition, the nuclei of 500 cells were each visually scored and classified into five categories corresponding to the amount of DNA in the tail (Stages A–E with <5%, 5–20%, 20–40%, 40–95%, and >95% DNA in tail, respectively). The tailfactor (*tf*) was calculated with the following formula:

$$tf\ (\%\text{DNA in tail}) = \left(\frac{A*F(A)+B*F(B)+C*F(C)+D*F(D)+E*F(E)}{(A+B+C+D+E)}\right)$$

where A–E are the numbers of cells classified into a stage group and F(A–E) the theoretical average of each category (2.5, 12.5, 30, 67.5, 97.5%). Differences between sham and exposed cells were analyzed statistically with the paired Student´s *t*-test to compare the percentage of cells for each category and the *tf* values of all experimental replicates. For each experiment, additionally, the difference in Comet stage category distribution between sham and exposed cells was tested for statistical significance with Pearson's chi-squared (χ^2). The indicated numbers are mean *p*-values for all experiments.

S1.5. Sister chromatid exchange (SCE) assay

The SCE assay was made for HTR-8/SVneo cells according to standard protocols [34] with some modifications. The population doubling time of these cells is about 24 h; accordingly, differential staining of chromatids was achieved by culturing them in the presence of 10 µM BrdU for 64 h while

preventing light exposure. Cells were seeded into 35-mm Petri dishes (Nunc) at a density of 1.7 ×10^4 cells/cm^2 and grown in the presence of BrdU for 24 h. Then, they were subjected to 24 h of exposure to wEMF, followed by a recovery phase of 16 h, each time with exchange of media and addition of 2 µM PARP inhibitor AG-014699 (Selleck Chemicals) when indicated. Finally, cells were arrested in metaphase by the addition of 40 µL colcemid solution (HybriMax, Gibco) to 3 mL of medium and incubation for 1.5 h, harvested by trypsinization, and washed with PBS. Cells were then carefully resuspended in 14 mL of prewarmed hypotonic solution (5.6 g/L KCl) and swelled at 37°C for 20 min. After addition of 1 mL freshly prepared chilled fixative (methanol/acetic acid 3:1), cells were pelleted at 650×g for 10 min and washed 3 × with decreasing volumes of cold fixative, then finally resuspended in 200–250 µL of fixative. Three drops of the cells suspension was spread on clean microscopic slides, rinsed with fixative, air dried, and aged at 37°C for 3 days. Slides were then stained with 0.5 mg/L Hoechst 33258 for 15 min, rinsed, submerged in 2× SSC, exposed to 5 J/cm^2 UV-B (Bio-Sun, Vilber Lourmat), rinsed with ddH_2O, and air dried. Finally, slides were stained with 10% Giemsa staining solution (Merck Millipore), rinsed with ddH_2O, air dried, and mounted with Entellan (Merck Millipore). Blinded for the examiner, images of metaphase spreads were recorded at 100× magnification on a Zeiss Axiophot microscope, and the numbers of SCE, break points, and chromosomes per cell were counted. Pooled SCE data and means of the indicated number of independent experimental replica were statistically analyzed with ANOVA and the Student's *t*-test, respectively, with the GraphPad PRISM 8 software package.

S1.6. Live cell imaging of XRCC1 recruitment

For live-cell imaging, U-2 OS cells were seeded on round cover slips (ø 18 mm) at a density of 2.5 x10^4 cells/cm^2. The coverslip was then mounted into the exposure chamber sXcli-2450 for live-cell imaging. The setup was then inserted on a Leica 6000B epifluorescence microscope equipped with a temperature-controlled incubation chamber and an UV-A laser at 355 nM (Q-switched CristaLaser). In the absence of photosensitizers and applied at moderate doses, this UV-A laser can be used to induce local oxidative DNA damage and single-strand breaks [35]. The amount of induced DNA damage correlates with the irradiated area and time as well as the laser intensity. To minimize inter-experimental variation, constant areas of 1'500 (15x100) and 2'500 (25×25) pixels at 100× magnification were irradiated with the UV-A laser (pixel size 5) for 5 and 10 ms to assess short- and long-term recruitment dynamics, respectively. Importantly, the laser power was calibrated regularly to reduce variation of the amount of induced DNA damage. Randomly chosen cells were irradiated and imaged sequentially with the indicated time intervals using low intensity exposure and illumination settings (250 ms and 30% FIM, respectively). To avoid any experimenter bias, sham or wEMF exposure was always performed blinded for the observer and all image processing and quantitation were done in an automated way using the versatile open source software "CellProfiler 2.0" (http://www.cellprofiler.org) [36]. For each picture, the software recognized and quantified the nucleus as well as the damaged region by the local increase of the pixel intensities. Relative XRCC1 recruitment for each assessed cell was calculated by dividing the median pixel intensities of the site of DNA damage with the median pixel intensity of the nucleus. Based on the data of the indicated number of assessed nuclei from several independent experiments, the recruitment curves were statistical analyzed by two-way ANOVA for repeated measurement and post-hoc Bonferroni test for matched time-points using the GraphPad PRISM 8 software.

S2. Supplementary Figures

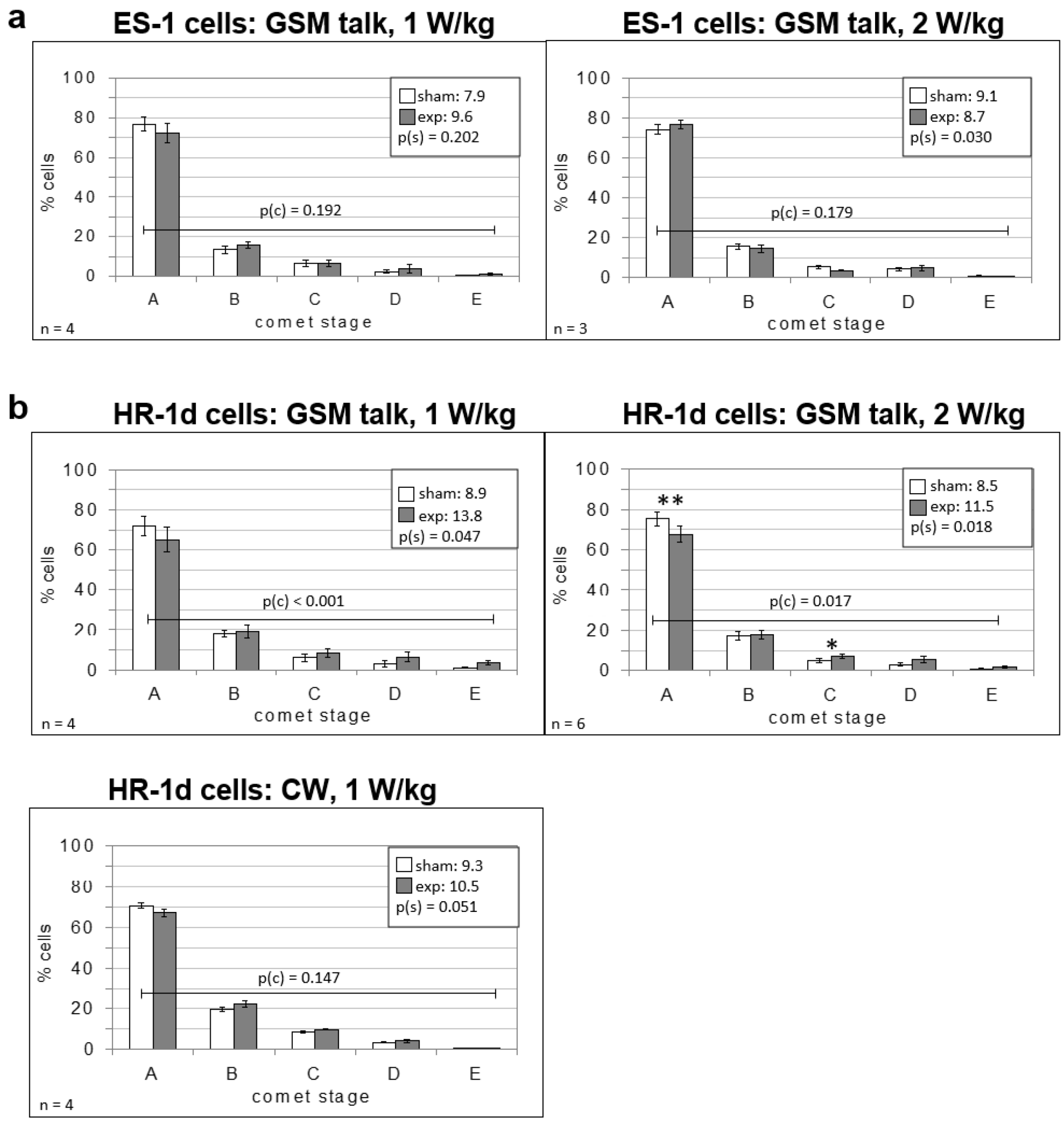

Figure S15. Results of CAs by visual scoring of wEMF-exposed primary human fibroblasts. (**a**) ES-1 or (**b**) HR-1d cells were exposed to intermittent (5/10 min on/off) GSM-talk modulated or unmodulated (CW) wEMF for 16 h. Percentages of cells in comet stages A to E, classified by visual scoring, are displayed. n, number of experiments; error bars, SEMs; statistical significance was assessed by the Student´s *t*-test comparing individual comet stage categories, * p<0.05; ** p<0.01; *** p <0.001. The horizontal bar and p(c) show the mean significance of χ^2 tests analyzing the comet stage distribution of sham and wEMF-exposed nuclei of individual experiments. Upper right boxes show the mean tailfactor for sham and exposed cells as well as the significance level of the Student´s *t*-test on the tailfactors (p(s)).

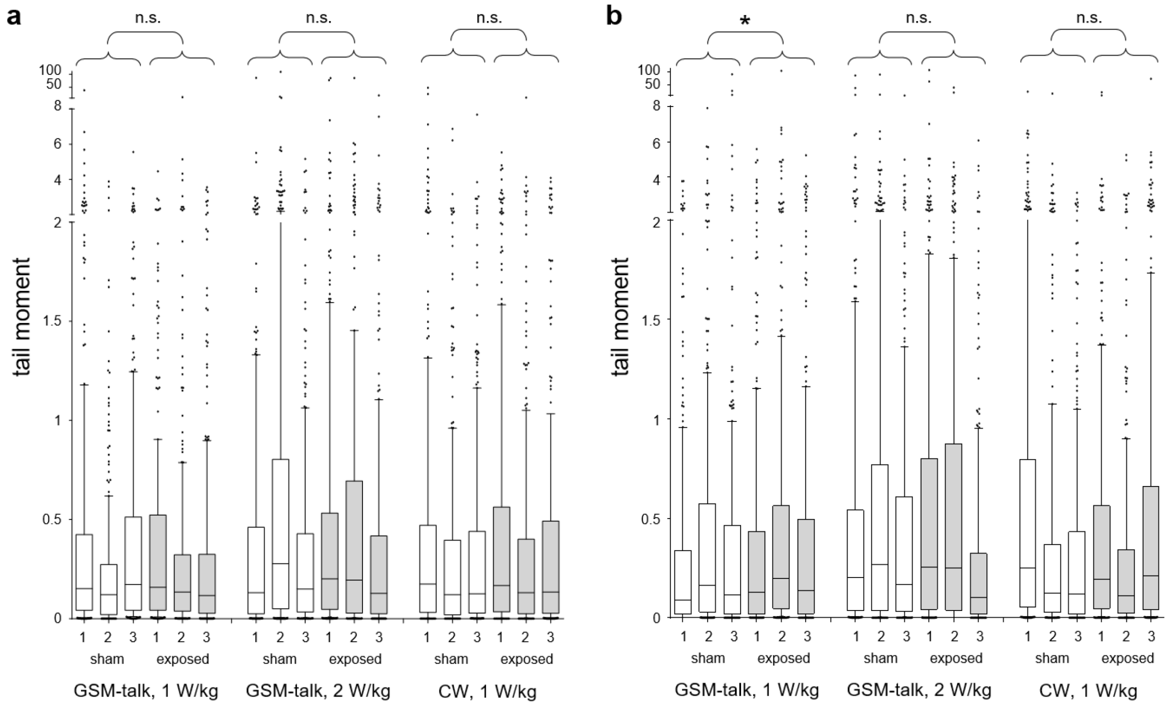

Figure S16. Results of automatically scored CAs of primary human fibroblasts exposed to GSM-talk and unmodulated RF-EMF. (**a**) ES-1 or (**b**) HR-1d cells were exposed to intermittent (5/10 min on/off) RF-EMF for 16 h. Displayed are the medians of automated analysis of pooled tail moment values from 300 cells (100 cells from 3 slides) per condition for 3 independent experiments per exposure condition. Boxes and whiskers indicated interquartile range and 10–90 percentile range, respectively. Statistical significance is according to Student´s *t*-tests comparing the medians of each single experiment: * $p<0.05$; ** $p<0.01$; *** $p<0.005$.

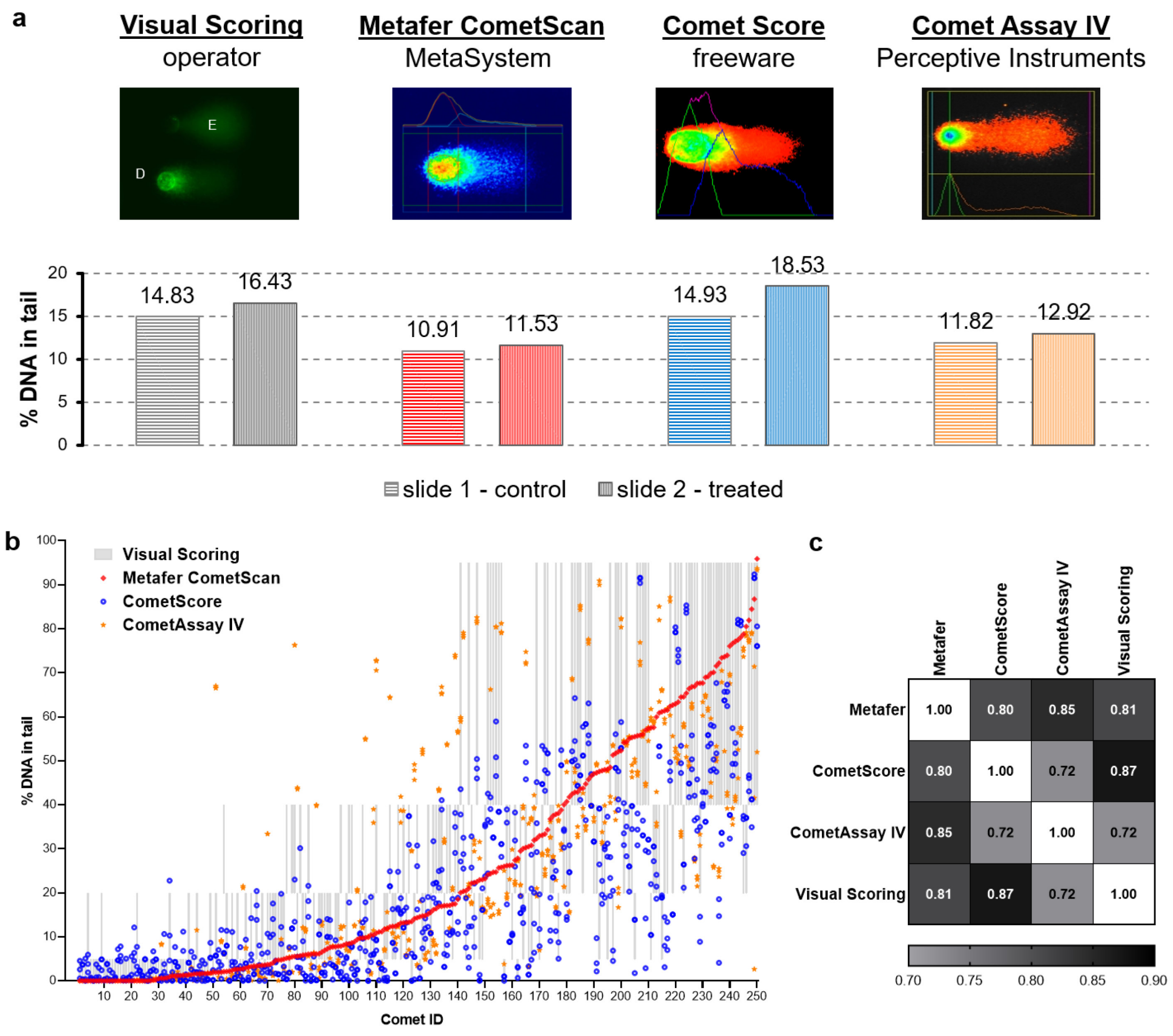

Figure S17. Comparison of Comet scoring methods. (**a**) Two randomly chosen electrophoresed slides with MRC-5 fibroblast cells, either untreated (control) or treated with 10 µM H_2O_2 for 15 min (treated) were analyzed by operator-based visual scoring, automated Comet recognition and analysis (Metafer CometScan), and semi-automated quantification software (CometScore and Comet Assay IV) of operator-selected nuclei. The mean "% DNA in tail" of about 400–450 analyzed nuclei is shown. (**b**,**c**) A test set of 250 Comets ranging from 0–95% of DNA migrating out of the nucleus was obtained from control and H_2O_2-treated slides and analyzed by the different scoring methods. Ranked by the readout of Metafer CometScan, the repeated measurements/categorization are blotted (**b**) and analyzed by Spearman correlation (**c**) in GraphPad Prism 8, which confirmed highly significant correlation between all methods and *r* values above 0.72.

a

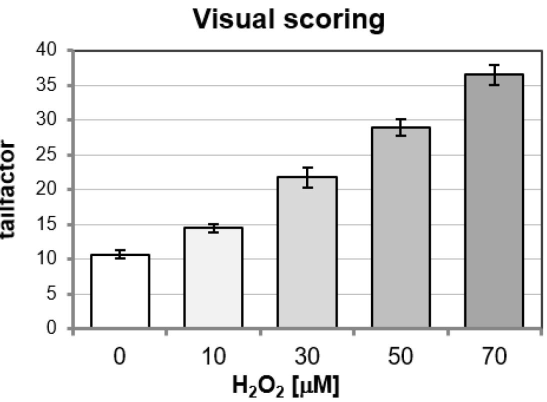

H_2O_2 [µM]	n	replica			Ø tail-factor	*p*-value *t*-test	
		1	2	3		paired	Welch
0	2318	13.04	10.58	8.46	10.69		
10	2586	16.20	15.42	11.75	14.46	0.020	0.119
30	2145	24.33	25.08	15.73	21.72	0.034	0.050
50	1977	31.70	31.25	23.87	28.94	0.007	0.008
70	1572	38.67	40.54	30.20	36.47	0.008	0.007

b

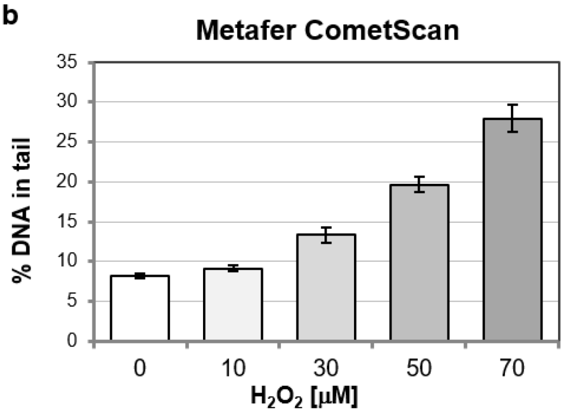

H_2O_2 [µM]	n	replica			Ø DNA in tail	*p*-value *t*-test	
		1	2	3		paired	Welch
0	2119	9.49	7.99	7.05	8.18		
10	2313	10.62	8.65	8.21	9.16	0.026	0.394
30	2110	14.80	15.99	9.17	13.32	0.094	0.123
50	1899	22.74	20.32	15.98	19.68	0.013	0.019
70	1503	30.76	32.27	20.92	27.98	0.024	0.027

c

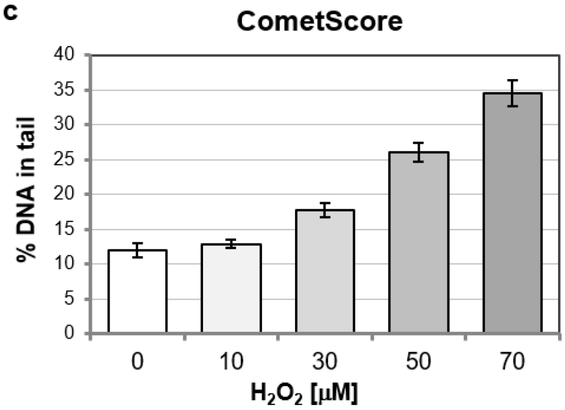

H_2O_2 [µM]	n	replica			Ø DNA in tail	*p*-value *t*-test	
		1	2	3		paired	Welch
0	2618	14.74	13.48	7.73	11.98		
10	2900	15.14	12.79	10.73	12.88	0.497	0.742
30	2642	19.57	19.90	13.73	17.73	0.007	0.123
50	2283	31.09	26.05	21.10	26.08	0.007	0.020
70	1882	35.11	40.88	27.39	34.46	0.012	0.014

Figure S18. Comparison of the sensitivity of Comet scoring methods. MRC-5 cells were exposed to the indicated concentrations of H_2O_2 for 10 min. The level of DNA damage was assessed by the alkaline Comet assay and the same set of Comets were scored by (**a**) visual classification, (**b**) by automated analysis with the Metafer CometScan software and (**c**) by manual selection of nuclei and quantification with the freeware CometScore. Shown are the mean of 3 independent biological experiments with indicated number of analyzed nuclei (n), statistically analyzed in GraphPad Prism by paired or Welch's corrected Student's *t*-test.

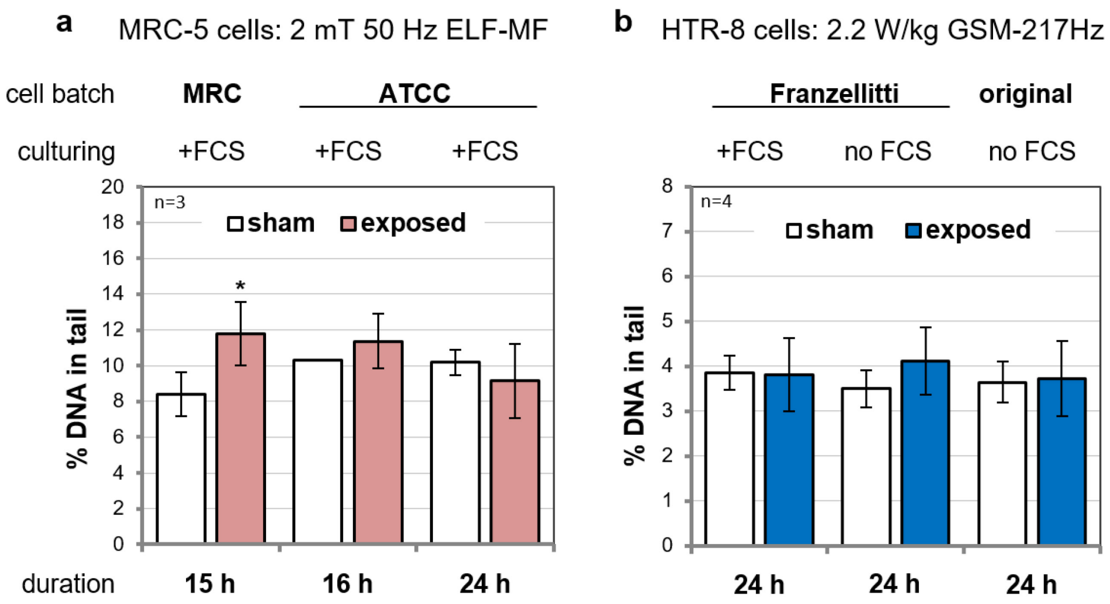

Figure S19. Cell batch and culturing condition as possible confounder of exposure effect in CAs. (**a**) Primary MRC-5 fibroblasts from early passage obtained directly from the Medical Research Council UK (MRC) and used in Focke *et al.* [37] and later passage cell batches from stock center (ATCC) were intermittent (5/10 min on/off) exposed to 50 Hz ELF-MF with powerline harmonics for the indicated period. Applying alkaline CA with automated analysis, median fragmentation of nuclear DNA was measured. (**b**) Batches of immortalized HTR-8/SVneo (HTR-8) trophoblast cells were obtained from Franzellitti *et al.* [22] and the original source [21]. During intermittent exposure (5/10 min on/off) with GSM-217Hz modulated wEMF (SAR 2.2 W/kg) for 24 h, cells were kept in media either with or without supplemented FCS (10%). Mean DNA damage level in alkaline CA were calculated for each experimental condition. Error bars indicate the SEM and asterisks represent the significance levels Student´s *t*-test for paired values of independent experiments (n≥3): * $p<0.05$.

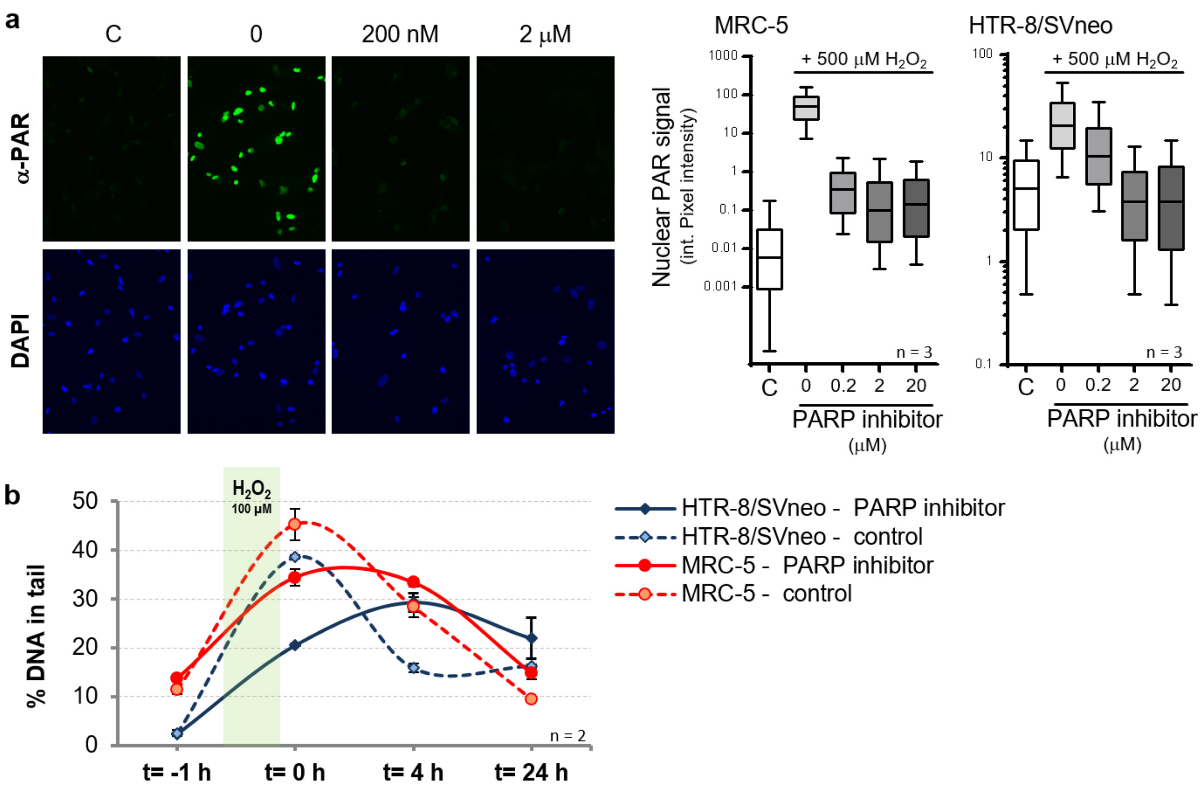

Figure S20. Changing BER dynamics by pharmacological inhibition of PARP1. (**a**) MRC-5 and HTR-8/SVneo cells were treated with increasing concentration of the PARP inhibitor AG-014699 for 24 h, prior to induction of oxidative DNA damage with 500 µM H_2O_2 and subsequent analysis of nuclear poly(ADP-ribose) (PAR) synthesis by immunofluorescence. Depicted are representative pictures of immunofluorescence for PAR and nuclear counterstaining with DAPI in MRC-5 cells and the pooled data of PAR quantification from 3 independent experiments. C: PAR levels of unchallenged cells. Boxes represent median, 10% and 90% percentiles; whiskers indicate minimum and maximum values. (**b**) Assessment of DNA repair kinetics by alkaline CA in cells pre-treated with 2 µM of PARP inhibitor AG-014699 for 16 h prior to induction of oxidative DNA damage with 100 µM H_2O_2 for 30 min (t = 0 h).

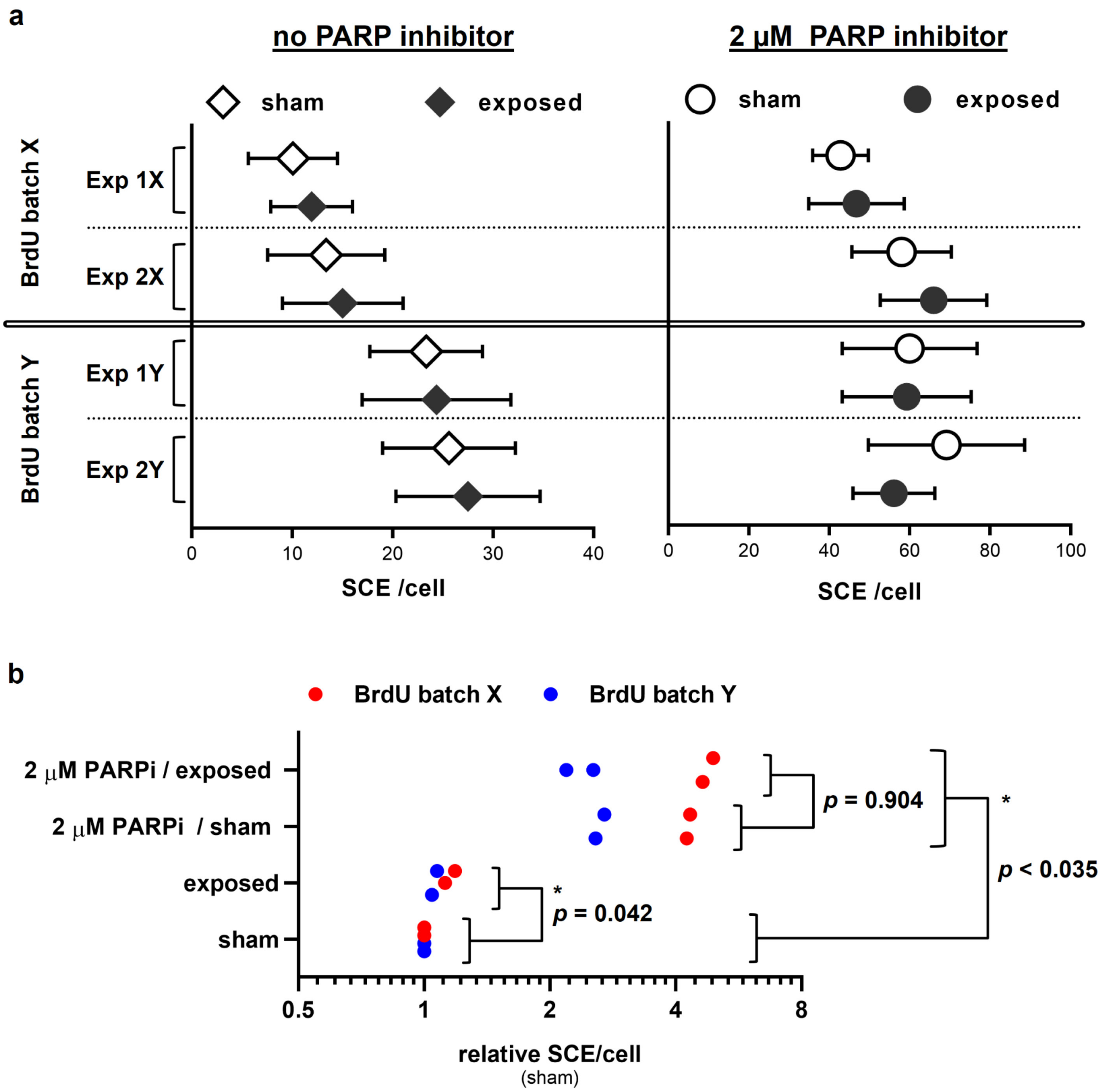

Figure S21. UMTS exposure affects the rate of sister chromatid exchange. Human trophoblast HTR-8/SVneo cells were cultured in the presence of 10 µM BrdU (two different batches used) for two cell cycles. During the second cell cycle, cells were exposed to an intermittent (5/10 min on/off) UMTS signal at 4.92 W/kg SAR for 24 h in presence or absence of 2 µM of PARP inhibitor AG-014699, after which sister chromatid exchanges (SCE) were analyzed. (**a**) Shown are means and standard deviations (SD) of the number of SCE/cell of 4 independent experiments grouped by BrdU batches used. (**b**) Upon normalization to the respective sham-exposure condition, statistical analysis by Student's *t*-test with Welch's correction were performed and *p*-values are indicated.

S3. Supplementary References

21. Graham, C.H.; Hawley, T.S.; Hawley, R.G.; MacDougall, J.R.; Kerbel, R.S.; Khoo, N.; Lala, P.K. Establishment and characterization of first trimester human trophoblast cells with extended lifespan. Exp. Cell Res. 1993, 206, 204-211, doi:10.1006/excr.1993.1139.
22. Franzellitti, S.; Valbonesi, P.; Ciancaglini, N.; Biondi, C.; Contin, A.; Bersani, F.; Fabbri, E. Transient DNA damage induced by high-frequency electromagnetic fields (GSM 1.8 GHz) in the human trophoblast HTR-8/SVneo cell line evaluated with the alkaline comet assay. Mutat. Res. 2010, 683, 35-42, doi:10.1016/j.mrfmmm.2009.10.004.
24. Dobkin, D.M. The RF in RFID: Passive UHF RFID in Practice, 1st ed.; Elsevier Burlington, 2008; pp. 1-504, doi:10.1016/B978-075068209-1.50015-2.
25. EPCglobal. EPC Radio-Frequency Identity Protocols Class-1 Generation-2 UHF RFID. (v1.2.0). 2008, https://www.gs1.org/sites/default/files/docs/epc/uhfc1g2_1_2_0-standard-20080511.pdf.
26. Andersen, J.B.; Mogensen, P.E.; Pedersen, G.F. Power variations of wireless communication systems. Bioelectromagnetics 2010, 31, 302-310, doi:10.1002/bem.20569.
27. Schuderer, J. EMF risk assessment: "in vitro" research and sleep studies. Doctoral Thesis, ETH Zürich, Zurich, 2004, doi:10.3929/ethz-a-004752056.
28. Verloock, L.; Joseph, W.; Vermeeren, G.; Martens, L. Procedure for assessment of general public exposure from WLAN in offices and in wireless sensor network testbed. Health Phys. 2010, 98, 628-638, doi:10.1097/HP.0b013e3181c9f372.
29. IEEE. IEEE Std 802.11g-2003, Part 11: Wireless LAN Medium Access Control (MAC) and Physical Layer (PHY) Specifications, Amendment 4: Further Higher Data Rate Extension in the 2.4 GHz Band. 2003, pp 1-104, doi:10.1109/IEEESTD.2003.94282.
30. Singh, N.P.; McCoy, M.T.; Tice, R.R.; Schneider, E.L. A simple technique for quantitation of low levels of DNA damage in individual cells. Exp. Cell Res. 1988, 175, 184-191, doi:10.1016/0014-4827(88)90265-0.
31. Anderson, D.; Yu, T.W.; Phillips, B.J.; Schmezer, P. The effect of various antioxidants and other modifying agents on oxygen-radical-generated DNA damage in human lymphocytes in the COMET assay. Mutat. Res. 1994, 307, 261-271, doi:10.1016/0027-5107(94)90300-x.
32. Ivancsits, S.; Diem, E.; Pilger, A.; Rudiger, H.W.; Jahn, O. Induction of DNA strand breaks by intermittent exposure to extremely-low-frequency electromagnetic fields in human diploid fibroblasts. Mutat. Res. 2002, 519, 1-13, doi:10.1016/S1383-5718(02)00109-2.
33. Smith, C.C.; O'Donovan, M.R.; Martin, E.A. hOGG1 recognizes oxidative damage using the comet assay with greater specificity than FPG or ENDOIII. Mutagenesis 2006, 21, 185-190, doi:10.1093/mutage/gel019.
34. Sunada, S.; Haskins, J.S.; Kato, T.A. Sister Chromatid Exchange as a Genotoxic Stress Marker. In Radiation Cytogenetics: Methods and Protocols, Kato, T.A., Wilson, P.F., Eds. Springer New York: New York, NY, 2019; pp. 61-68.
35. Lan, L.; Nakajima, S.; Oohata, Y.; Takao, M.; Okano, S.; Masutani, M.; Wilson, S.H.; Yasui, A. In situ analysis of repair processes for oxidative DNA damage in mammalian cells. Proc. Natl. Acad. Sci. U.S.A. 2004, 101, 13738-13743, doi:10.1073/pnas.0406048101.
36. Lamprecht, M.R.; Sabatini, D.M.; Carpenter, A.E. CellProfiler: free, versatile software for automated biological image analysis. BioTechniques 2007, 42, 71-75, doi:10.2144/000112257.
37. Focke, F.; Schuermann, D.; Kuster, N.; Schär, P. DNA fragmentation in human fibroblasts under extremely low frequency electromagnetic field exposure. Mutat. Res. 2010, 683, 74-83, doi:10.1016/j.mrfmmm.2009.10.012.
48. Schwarz, C.; Kratochvil, E.; Pilger, A.; Kuster, N.; Adlkofer, F.; Rüdiger, H.W. Radiofrequency electromagnetic fields (UMTS, 1,950 MHz) induce genotoxic effects in vitro in human fibroblasts but not in lymphocytes. Int. Arch. Occup. Environ. Health 2008, 81, 755-767, doi:10.1007/s00420-008-0305-5.
